# A Pilot Study on the Efficacy of a Single Intra-Articular Administration of Triamcinolone Acetonide, Hyaluronan, and a Combination of Both for Clinical Management of Osteoarthritis in Police Working Dogs

**DOI:** 10.3389/fvets.2020.512523

**Published:** 2020-11-06

**Authors:** João C. Alves, Ana Santos, Patrícia Jorge, Catarina Lavrador, L. Miguel Carreira

**Affiliations:** ^1^Divisão de Medicina Veterinária, Guarda Nacional Republicana (GNR), Lisbon, Portugal; ^2^MED—Mediterranean Institute for Agriculture, Environment and Development, Instituto de Investigação e Formação Avançada, Universidade de Évora, Évora, Portugal; ^3^Faculty of Veterinary Medicine, University of Lisbon (FMV/ULisboa), Lisbon, Portugal; ^4^Interdisciplinary Centre for Research in Animal Health (CIISA), University of Lisbon (FMV/ULisboa), Lisbon, Portugal; ^5^Anjos of Assis Veterinary Medicine Centre (CMVAA), Barreiro, Portugal

**Keywords:** animal model, osteoarthritis, pain, triamcinolone, hyaluronan

## Abstract

**Objectives:** To describe and compare the use and effectiveness of a single intra-articular injection (IA) of triamcinolone acetonide (TA), hyaluronan (HA), and a combination of both (TA+HA) in police working dogs with natural occurring hip osteoarthritis (OA).

**Study Design:** Prospective, randomized, single-blinded study.

**Sample Population:** Thirty animals with naturally occurring hip OA.

**Methods:** Animals were randomly divided in three groups: GT, treated with 20 mg of TA per hip joint; GH, treated with treated 20 mg of HA per hip joint; and GTH, treated with a combination of 20 mg of TA and 20 mg of HA per hip joint. Response to treatment, measured by the Canine Brief Pain Inventory (divided in Pain Interference Score—PIS and Pain Severity Score—PSS) and the Hudson Visual Analog Scale (HVAS), was evaluated in seven different time points: T0 (before treatment), T1 (after 15 days), T2, T3, T4, T5, and T6 (after 1, 2, 3, 4, and 5 months, respectively). Results were compared using a Kruskal-Wallis test or a Wilcoxon signed ranks test, and *p* < 0.05 was set.

**Results:** Comparing results of the different time points considered with T0, significant differences were registered in GH at T1 for HVAS (*p* = 0.03) and PIS (*p* = 0.04); and in GTH at T1 (*p* = 0.05 for HVAS and *p* < 0.05 for PIS), T2 (*p* < 0.04 for PIS), T3 (*p* < 0.03 for HVAS and *p* = 0.05 for PIS), T4 (*p* < 0.03 for HVAS and *p* < 0.05), and T5 (*p* < 0.05 for HVAS). No significant differences were found between groups when comparing scores in each time point. Individual treatment is considered successful with a reduction of ≥1 for PSS or ≥2 for PIS. In GTH, treatment was successful in four animals between T1 and T5 (40%, *n* = 10) and three at T6–T7 (30%, *n* = 10) for PSS and three animals of GTH at T1 (30%), two at T2 (20%), three between T3 and T4 (30%), and two between T5 and T7 (20%).

**Conclusions and Clinical Relevance:** This study provides direct information on the use of these treatment modalities in patients with hip OA. Intra-articular injection with TA and HA may be a treatment option for dogs with naturally occurring OA, particularly when simultaneously used, as they provide significant improvements of PIS and HVAS scores. Individual scores improved in some animals with PIS, PSS, and HVAS.

## Introduction

Osteoarthritis (OA) is a complex joint disease with a high negative impact on patient's quality of life and a high financial burden. Characterized by its inflammatory character and degradation of cartilage layers, it is a source of chronic pain, which affects all mammals, including humans and dogs (1–3). In adult active dogs, OA presents a prevalence around 20% (4–6). This value is expected to rise, due to a simultaneous increase in life expectancy and obesity. Both surgical and natural occurring canine models have been widely studied, and since pathologic process, clinical presentation and response to treatment are very similar in both species—humans and dog, this animal model is the closest to a gold standard (7–10). The changes that occur in slowly progressive spontaneous dog OA closely match those of human OA, in contrast with those seen in rapidly advancing experimental surgical induced OA (11). The grades of canine hip OA are similar to those in the classification of human OA (mild/minimal, moderate, and severe) (12, 13). In addition, companion animals share the same environment and suffer similar co-morbidities as humans, with OA usually being present for prolonged periods. Therefore, these naturally occurring painful disease models may better reflect the complex genetic, environmental, temporal, and physiological influences present in humans (12). Exploring spontaneous dog OA under the One Medicine concept can promote new insight on the disease, improving therefore the health and well-being of both species, humans, and dogs (12, 13).

Intra-articular (IA) corticosteroids (CS) have been used for several decades in humans and horses to successfully palliate pain and control inflammation associated with OA and surrounding tissues (14, 15). Different guidelines for the management of human OA provide varying strength of recommendation for the use of intra-articular CS, from weak to strong recommendation (16–20). On the other hand, other guidelines state an inability to recommend for or against the use of intra-articular corticosteroids, in this case specifically for patients with symptomatic knee OA (21). Corticosteroids reduce the number of inflammatory cells such as lymphocytes, macrophages and mast cells, and also slow down the synthesis of inflammatory mediators such as interleukin 1β, Tumor necrosis factor α, and Cycloxygenase 2 in the synovial fluid (22–25). The pain relief they provide is attributed to the inhibition of prostaglandin synthesis (24). Triamcinolone is recommended over other CS due to an extended duration of action (26, 27). Hyaluronan (HA), the high molecular glycosaminoglycan, occurs naturally in synovial fluid, and provides joint lubrication, helps limit inflammation, pain and cartilage degradation while acting as a shock absorber, allowing the joint to move in a smooth manner (16, 17). Its mechanism of action is not completely understood, but anti-inflammatory, anti-nociceptive, and chondroprotective properties have been suggested, through the enhancement of cartilage synthesis, blunting response to interleukin 1, protection from the damage of oxygen free radicals, and protection of chondrocytes from apoptosis (28–31). Guidelines for the management of OA provide a weak recommendation for the use of IA HA (20), a conditional recommendation against (19) or that they should not be offered as an option (18). The choice to use IA HA or a CS, or which CS to use, is often determined by individual preference of the clinician (18, 19). A popular approach is their combined administration, thus providing rapid onset of action (obtained from the CS), with prolonged effect and decreasing the potential side effects of intra-articular CS therapy (obtained from HA) (20, 21). Being an incurable chronic disease, treatment success in OA is often defined as the ability to manage its symptoms, mainly pain. The similarities in neurophysiology across mammals strongly suggest that the type of pain experienced by humans and animals is analogous (22). The Canine Brief Pain Inventory (CBPI) was developed to assess the impact of chronic pain in the patient's life. It is divided in two sections, a pain severity score (PSS) that assesses the magnitude of the animal's pain, and a pain interference score (PIS) that assesses the degree in which pain affects daily activities (23). The Hudson Visual Analog Scale (HVAS) has been validated for the assessment of mild to moderate lameness in dogs, using force plate analysis as a criterion-reference standard (24).

With this study, we aimed to determine (1) if the intra-articular administration of triamcinolone acetonide (TA) or HA can reduce pain scores in a naturally occurring canine osteoarthritis model and if (2) the combined administration of both substances provides better results for longer periods of time.

## Methods

The study used a sample of 30 working dogs (*N* = 30) from the *Guarda Nacional Republicana* (Portuguese Gendarmerie Canine Unit) of both genders (6 females and 24 males), with a mean age of 6 ± 2.4 years old and body weight of 33.3 ± 6.65 kg. Breeds included German Shepherd Dogs (*n* = 20), Belgian Malinois Shepherd Dogs (*n* = 5), Labrador Retriever (*n* = 4), and Dutch Shepherd Dog (*n* = 1). All had bilateral naturally occurring mild and moderate hip OA, classified according to the Orthopedic Foundation for Animals scoring, This method was chosen due to the unavailability of other evaluation methods, such as PennHip. Radiographic evaluation was performed by one of the authors (JCA), not a board radiologist but with extensive training and experience in radiographic examination.

Patients were included based on trainer complaints, physical examination, and standard pelvis radiographic evaluation consistent with bilateral hip OA. Animals with other diseases were ruled out through physical examination, complete blood count, and basic serum chemistry profile (BUN, Creat, ALT, AST, Gluc) were not included in the study. Patients under any treatment, therapy or supplement were also excluded. Written, informed consent was obtained from the Institution responsible for the animals.

Dogs were randomly divided in three groups according to the type of drug used for hip joint IA administration, namely: GT (treated with 20 mg of TA per hip joint—Trigon depot, Bristol-Myers Squibb®, Spain), GH (treated with 20 mg of hyaluronan per hip joint—Hyalart, Grunenthal®, Portugal), and GTH (treated with the combination of both substances per hip joint). Breeds were similarly distributed amongst groups: GT had 7 German Shepherd Dogs, 2 Belgian Malinois Shepherd Dog and 1 Labrador Retriever; GH had 6 German Shepherd Dogs, 2 Belgian Malinois Shepherd Dogs, 1 Labrador Retriever and 1 Dutch Shepherd Dog; and GTH had 7 German Shepherd Dogs, 1 Belgian Malinois Shepherd Dogs and 2 Labrador Retrievers.

The IA administration was always made by the same clinician and conducted with dogs under light sedation using medetomidine (0.01 mg/kg) and buthorphanol (0.1 mg/kg), both administered intravenously, and with intravenous fluids of NaCl 0.9% in the dose of 2 ml/Kg/h. Animals were placed in lateral recumbency, and a small window of 4 cm × 4 cm area surrounding the greater trochanter was clipped and aseptically prepared, using a chlorhexidine solution 0.2% followed by 70% alcohol application, using sterile gloves and 10 cm × 10 cm gauzes. With the limb parallel to the table surface and in a neutral position, the operator inserted a 22-gauge with 75 mm length spinal needle, closely dorsal to the greater trochanter and perpendicular to the long axis of the limb (32). Confirmation of correct needle placement was obtained through the collection of synovial fluid. Both hips were treated with the same treatment in all animals. After treatment, animals were rested for 3 consecutive days. Signs of exacerbated pain during daily activities or physical examination (pain during joint mobilization, stiffness, and reduced range of motion), persistent stiffness of gait and changes in posture exhibited by the dogs, were evaluated by the veterinarian on the days 1 and 3 after the IA procedure. With IA treatments, some side effects were documented, and include local pain and inflammation, swelling and infection. These are usually self-limiting, and take 2–10 days to resolve (33, 34). If no complaints were registered, the animal could resume its normal activity over a period of 5 days (35, 36).

To evaluate the response to treatment and comparing it with the initial clinical condition, two validated tools for dog pain assessment were used: the CBPI (Appendix A) and the HVAS (Appendix B). These were completed by the trainers, who were unaware of which treatment the animal received. Evaluations were conducted at T0 (before IA treatment), T1 (15 days after IA treatment), T2, T3, T4, T5, T6, and T7 (1, 2, 3, 4, 5, and 6 months after IA treatment, respectively).

From all the sampled individuals, three dogs from the GT were excluded—two after T2 due to having developed unrelated medical conditions (one developed gastric dilatation volvulus and the other suffered a third phalanx avulsion), and one after T3 due to an inability to maintain medical follow-up. Data collected from these animals was considered up to the point of their exclusion. Data was analyzed with IBM SPSS Statistics version 20, and a significance level of *p* < 0.05 was set. Normality was accessed with a Shapiro-Wilk test and results of all groups in each time point considered were compared using a Kruskal-Wallis test. When comparing each time point with T0 within each group, a Kruskal-Wallis test or a Wilcoxon signed ranks test was used.

## Results

**Figure 1:**
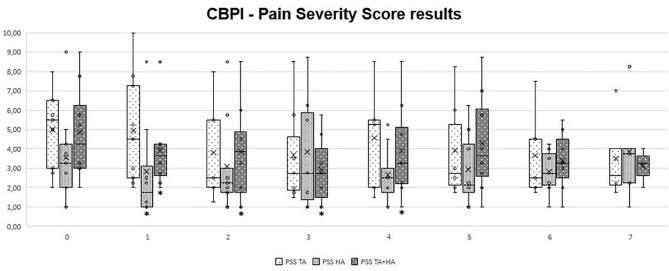
Overall Pain Severity Scores, by section and instant for triamcinolone acetonide (TA), hyaluronan (HA), and TA+HA. Box plots represent median, 25th and 75th percentiles, and whiskers represent 10th and 90th percentiles. *indicates significant differences within the group, when compared with T0.

**Figure 2:**
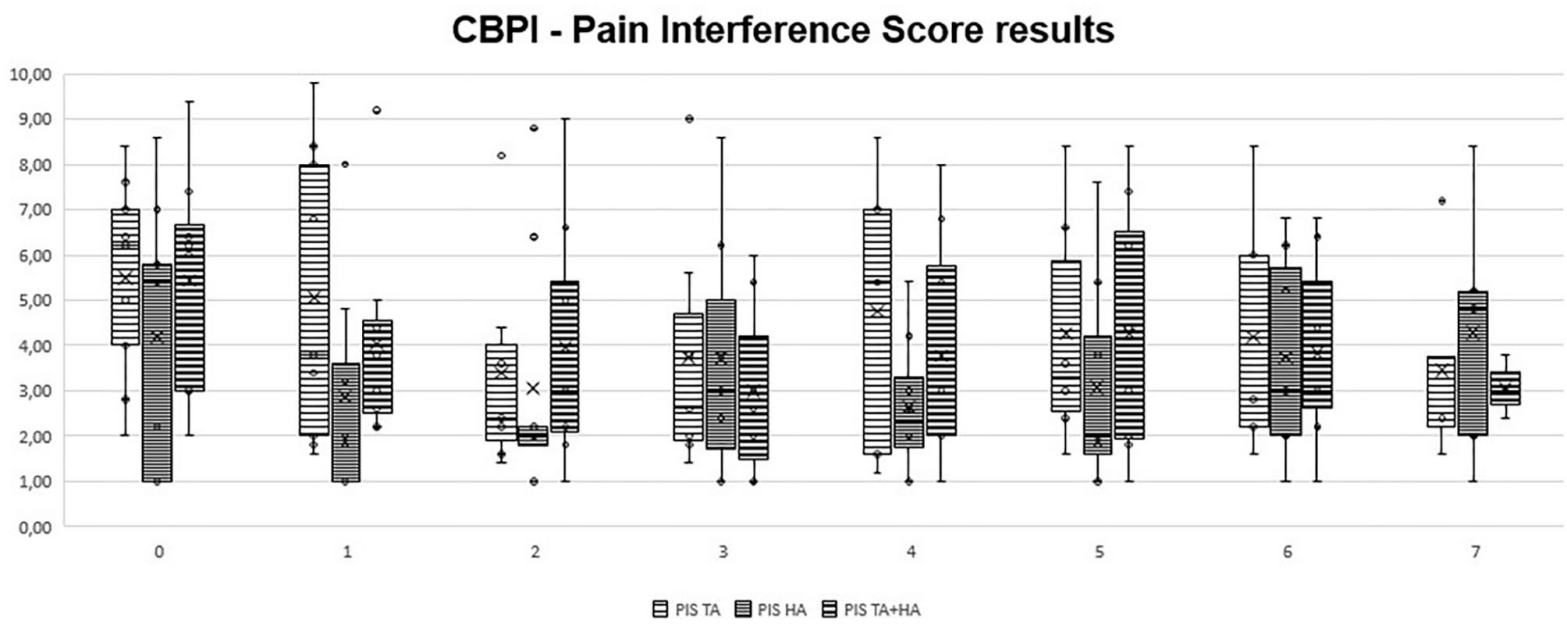
Overall Pain Interference Scores, by section and instant for triamcinolone acetonide (TA), hyaluronan (HA), and TA+HA. Box plots represent median, 25th and 75th percentiles, and whiskers represent 10th and 90th percentiles.

**Figure 3:**
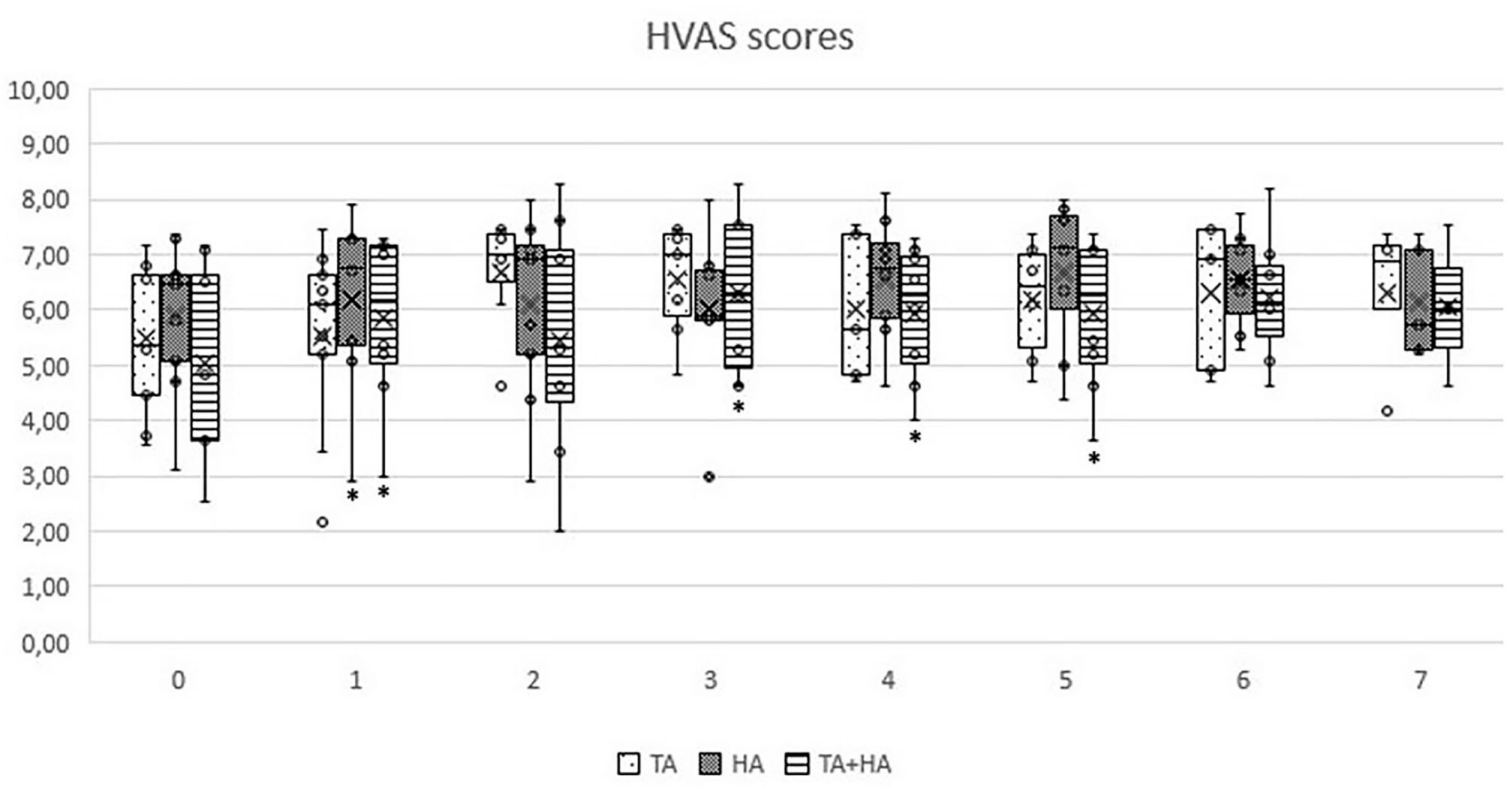
Overall Hudson Visual Analog scores, by instant for triamcinolone acetonide (TA), hyaluronan (HA), and TA+HA. Box plots represent median, 25th and 75th percentiles, and whiskers represent 10th and 90th percentiles. *indicates significant differences within the group, when compared with T0.

In GT, when comparing clinical results from T1 to T7 with patient initial condition (T0), no significant differences were recorded. In GH, significant differences were observed only at T1 (*p* = 0.03 for HVAS and *p* = 0.04 for PSS). In GTH, significant differences were observed at T1 (*p* < 0.05 for HVAS and *p* < 0.05 for PSS), T2 (*p* < 0.04 for PSS), T3 (*p* < 0.03 for HVAS and *p* < 0.05 for PSS), T4 (*p* < 0.03 for HVAS and *p* < 0.05 PSS), and T5 (*p* < 0.05 for HVAS). Comparing results of the three groups in each evaluation moment, no significant differences were found. Evolution of PSS, PIS, and HVAS scores can be observed in Figures 1–3, respectively. Evolution of mean PSS, PIS, and HVAS scores (±standard deviation), *p* values and percentage variations in each group, are presented in Table 1.

**Table 1:** Evolution of mean pain severity score (PSS) and pain interference score (PIS) of the Canine Brief Pain Inventory (CBPI), and improvements in HVAS scores (±standard deviation), by group and moment.

**Survey**	**Group**	**T0**		**T1**				**T2**				**T3**				**T4**				**T5**				**T6**				**T7**			
		**Score**	**SD**	**Score**	**SD**	***p***	**%**	**Score**	**SD**	***p***	**%**	**Score**	**SD**	***p***	**%**	**Score**	**SD**	***p***	**%**	**Score**	**SD**	***p***	**%**	**Score**	**SD**	***p***	**%**	**Score**	**SD**	***p***	**%**
PIS	GT	5.00	1.90	4.94	2.33	1.00	1.1	3.82	2.15	0.42	23.6	3.68	1.97	0.49	26.4	4.55	2.24	0.46	9.0	3.92	2.14	0.42	21.7	3.65	2.15	0.19	27.0	3.50	1.75	0.27	30.0
	GH	3.56	2.32	2.81	1.97	0.17	20.9	3.08	1.80	0.23	13.3	3.86	2.55	0.75	−8.5	2.66	1.11	0.87	25.3	2.94	1.61	0.79	17.4	2.82	1.08	0.68	20.6	3.85	1.82	0.36	−8.3
	GTH	4.88	2.35	3.91	1.34	0.18	19.9	3.84	2.03	0.21	21.2	2.93	1.42	0.06	39.9	3.91	1.95	0.16	19.9	4.31	2.14	0.53	11.5	3.39	1.48	0.27	30.4	3.08	0.72	1.00	36.8
PSS	GT	5.49	2.07	5.07	2.83	0.86	7.7	3.40	1.71	0.09	38.1	3.74	2.05	0.11	31.8	4.76	2.69	0.69	13.3	4.27	2.16	0.21	22.3	4.20	2.59	0.22	23.5	3.45	1.88	0.27	37.1
	GH	4.20	2.76	2.85	1.86	0.04	32.1	3.04	2.02	0.35	27.5	3.71	2.13	0.35	11.6	2.65	1.16	0.35	36.9	3.08	1.89	0.46	26.8	3.74	2.13	0.35	10.9	4.28	2.22	0.47	−1.9
	GTH	5.43	2.37	4.05	1.61	0.04	25.3	3.95	2.19	0.03	27.2	3.00	1.54	0.04	44.7	3.78	2.22	0.04	30.4	4.28	2.33	1.04	21.2	3.83	1.99	0.23	29.4	3.07	0.49	0.66	43.5
HVAS	GT	5.51	1.29	5.54	1.29	0.52	0.6	6.69	0.76	0.24	21.5	6.55	0.86	0.18	18.9	6.02	1.15	0.89	9.3	6.18	0.88	0.92	12.3	6.29	1.22	0.35	14.3	6.32	1.07	0.36	14.8
	GH	5.89	1.30	6.19	1.28	0.03	5.2	6.09	1.37	0.51	3.4	6.00	0.99	0.31	1.9	6.57	0.88	0.39	11.5	6.68	1.08	0.26	13.5	6.55	0.83	0.74	11.1	6.13	0.88	0.23	4.0
	GTH	5.03	1.63	5.85	1.31	0.04	16.4	5.44	1.62	0.16	8.2	6.31	1.27	0.03	25.5	5.95	1.01	0.03	18.4	5.94	1.22	0.04	18.2	6.23	1.10	0.11	24.0	6.06	0.99	0.11	20.5

During the study, no side effects were detected in any of the animals. All patients were able to resume normal activity after treatment.

## Discussion

OA is a chronic disease with no cure, but with the possibility to be effectively managed in a largely palliative approach, aiming to relieve symptoms and especially pain (17, 29). Non-steroidal anti-inflammatory drugs (NSAIDs) are often considered as the first line of OA treatment. For active patients, or with more advanced OA stages, the control they provide over signs and symptoms may be insufficient (30, 31, 37). Since OA is symptomatic only in the affected joint, while lacking obvious extra-articular manifestations, it is well-suited to have a local therapy administered by intra-articular injection, reducing the total amount required to produce an effect, compared with a systemic administration (38–40). Still, this approach presents some disadvantages, as the need of a precise diagnosis, the learning curve inherent to the execution of the procedure (particularly when considering hips), better conducted with the assistance of fluoroscopy or ultrasound, and the need of placing the patient under sedation or general anesthesia. Results showed that both TA and HA, when administered through intra-articular injection, are able to reduce pain levels to some degree and up to certain time points, in a naturally occurring canine osteoarthritis model. The combination of both substances, in particular, can be an effective therapeutic option, with a majority of treated patients showing improved results in their clinical condition, lasting for several months.

Limitations of this study are associated with sample size and the lack of a control group. Additionally, even though both the CBPI and HVAS have been validated as tools for the assessment of pain, lameness, and response to treatment in dogs, further studies should include another evaluation method such as Force Plait Gait or Stance Analysis.

The CBPI is often the analysis of choice in the veterinary literature, recommended for comparisons of pain scores between groups (41, 42). In this study, significant variations were observed in GH only at the first follow up, and for PSS. In GTH, improvements were also observed in PSS scores, but for a longer period. It was in GTH that the biggest improvement was observed, with a 44.7% improvement at T3. Individual treatment success has been set as a decrease in PSS ≥ 1 and in PIS ≥ 2 (28, 43). Both IA treatments with HA and TA were able to significantly reduce individual scores in naturally occurring canine OA model, particularly PSS, while improving the results for the majority of patients. In some cases of GT and in one patient of GH, beneficial results spread up to the last evaluation point, while most improvements in both groups declined around T4-T5. Results for PIS showed no significant variations, considering group results. Individual results registered some improvements, but less marked than PSS results. This may be because, for some patients, PIS scores were low to begin with, making it difficult to reach a significant reduction. In addition, these are dogs with very high prey drive and work motivation, which may lead to a good performance and low perception of pain interference during daily activities. Individual HVAS results improved in almost all animals. However, when considering group results, significant improvements were observed only in GH (at T1) and GTH, in this case up to T5. In GTH, similarly to PSS scores, improvements reached a highest of 25.5% at T3, and declined from this point, reaching an 18.2% improvement at T5. The IA use of HA for the treatment of OA is still somewhat controversial due to its mode of action being unclear and clinical trials have provided contradictory results (38). The results of our study showed improvements in some animals at T1, with the maximal number of significant improvements obtained at T2, and maintained for a couple of months. Additional administrations, compared to a single injection, may be required in order to obtain sustained results. IA CS have been used for several decades in humans to successfully palliate pain and control inflammation associated with OA and surrounding tissues (14, 15). A systematic review has deemed triamcinolone more effective in relieving pain and improving function than betamethasone and methylprednisolone acetate (44). The choice to use IA HA or CS, or which CS to use, is often determined by individual preference of the clinician (18, 19). There are reports presenting deleterious effects of IA CS, as they may induce the production of a low quantity and high viscosity synovial fluid. These results are often based on multiple injections, particularly of methylprednisolone, while a single dose does not seem to cause long-term detrimental effects (45, 46). In a canine model of OA, animals treated with IA triamcinolone showed a significant reduction of osteophyte size compared with a control group. At the histological level, it significantly reduced the severity of OA structural changes of cartilage and had no deleterious effects on normal cartilage (47). Our results show that a single IA TA is effective in reducing pain scores in some animals, but not the majority of them. For those that it was, benefits were detected for several months, in some cases up to the last evaluation point. We did not observed any clinical side-effects in the animals treated with TA, and this seems to be a safe therapeutic options in patients with hip OA. As we did not performed a follow-up radiographic evaluation of the joints, we cannot comment on the evolution of radiographic signs in the three considered groups, but it should be addressed in future studies.

Results observed in GTH support the hypothesis that combined administration of HA and TA is superior in positive effects when comparing to the individual use of each one. PSS scores show significant improvements until T3, raising from a 25.3% at T1 to a 44.7% improvement at T3. Individual PSS scores improved in several patients up to the six-month evaluation point. HVAS in GTH also improved significantly from T1 to T5, in contrast to what was observed in GT and GH. This result is in accordance with what was observed with PIS, reflecting increased mobility, presumably due to decreased pain.

According to the author's knowledge, this is the first study that presents the description of the clinical effect of IA CS, HA and the combined use of both products in a naturally occurring canine model. The study was able to establish that all therapeutic approaches are safe, since no side effects were observed after the IA procedure in all animals of the three groups considered. All treatments can be effective for the treatment of OA, particularly the combined use of both products (TA + HA). Future studies should enroll a larger sample and considered the effect of different doses and administration frequency.

## Conclusions and Clinical Relevance

Intra-articular administration of TA and HA may be a treatment option for natural occurring OA, particularly when used simultaneously. This study provides information on the use of these treatment modalities in patients with hip OA. Further studies are required, involving a larger number of patients and the use of a more objective evaluation method.

## Data Availability Statement

All datasets generated for this study are included in the article/Supplementary Material.

## Ethics Statement

This study is a part of a project approved by the ethical review committee of the University of Évora (Órgão Responsável pelo Bem-estar dos Animais da Universidade de Évora, approval no. GD/32055/2018/P1, September 25, 2018). Written informed consent was obtained from the owners for the participation of their animals in this study.

## Author Contributions

JA designed the protocol, conducted treatments, and prepared the manuscript. PJ and AS selected patients and conducted treatments. CL and LC revised the protocol and prepared the manuscript. All authors contributed to the article and approved the submitted version.

## Conflict of Interest

The authors declare that the research was conducted in the absence of any commercial or financial relationships that could be construed as a potential conflict of interest.

## References

[1] Loeser RF Goldring SR Scanzello CR Goldring MB. Osteoarthritis: a disease of the joint as an organ. Arthritis Rheum. (2012) 64:1697–707. 10.1002/art.34453 22392533 PMC3366018

[2] Venable RO Stoker AM Cook CR Cockrell MK Cook JL. Examination of synovial fluid hyaluronan quantity and quality in stifle joints of dogs with osteoarthritis. Am. J. Vet. Res. (2008) 69:1569–73. 10.2460/ajvr.69.12.1569 19046002

[3] Anderson KL O'Neill DG Brodbelt DC Church DB Meeson RL Sargan D. Prevalence, duration and risk factors for appendicular osteoarthritis in a UK dog population under primary veterinary care. Sci. Rep. (2018) 8:5641. 10.1038/s41598-018-23940-z 29618832 PMC5884849

[4] Allan GS Radiographic signs of joint disease in dogs and cats. In: Thrall DE editor. Textbook of Veterinary Diagnostic Radiology. 5th ed St. Louis, MO: Saunders Elsevier (2007). p. 317–58.

[5] Innes JF Arthritis. In: Tobias KM Johnson SA editors. Veterinary Surgery: Small Animal. St. Louis, MO: Elsevier Saunders (2012). p. 1078–111.

[6] Berenbaum F. Osteoarthritis as an inflammatory disease (osteoarthritis is not osteoarthrosis!). Osteoarthr. Cartilage. (2013) 21:16–21. 10.1016/j.joca.2012.11.012 23194896

[7] Kraus VBB Huebner JLL DeGroot J Bendele AM McIlwraith CW Frisbie DD The OARSI histopathology initiative – recommendations for histological assessments of osteoarthritis in the dog. Osteoarthr. Cartilage. (2010) 18:S66–79. 10.1016/j.joca.2010.04.015 20864024

[8] Gregory MH Capito N Kuroki K Stoker AM Cook JL Sherman SL. A Review of translational animal models for knee osteoarthritis. Arthritis. (2012) 2012:764621. 10.1155/2012/764621 23326663 PMC3541554

[9] Marijnissen ACA van Roermund PM TeKoppele JM Bijlsma JWJ Lafeber FPJG The canine “groove” model, compared with the ACLT model of osteoarthritis. Osteoarthr. Cartilage. (2002) 10:145–55. 10.1053/joca.2001.0491 11869074

[10] McCoy AM. Animal models of osteoarthritis: comparisons and key considerations. Vet. Pathol. (2015) 52:803–18. 10.1177/0300985815588611 26063173

[11] Liu W Burton-Wurster N Glant TT Tashman S Sumner DR Kamath RV. Spontaneous and experimental osteoarthritis in dog: similarities and differences in proteoglycan levels. J. Orthop. Res. (2003) 21:730–7. 10.1016/S0736-0266(03)00002-0 12798075

[12] Kohn MD Sassoon AA Fernando ND. Classifications in brief: Kellgren-Lawrence classification of osteoarthritis. Clin. Orthop. Relat. Res. (2016) 474:1886–93. 10.1007/s11999-016-4732-4 26872913 PMC4925407

[13] Puckler K Tellhelm B Kirberger R The hip joint and pelvis. In: Kirberger R McEvoy F editors. BSAVA Manual of Canine and Feline Musculoskeletal Imaging. New York, NY: Wiley (2016). p. 212–31.

[14] Lascelles BDX Brown DC Maixner W Mogil JS. Spontaneous painful disease in companion animals can facilitate the development of chronic pain therapies for humans. Osteoarthr. Cartilage. (2018) 26:175–83. 10.1016/j.joca.2017.11.011 29180098

[15] Meeson RL Todhunter RJ Blunn G Nuki G Pitsillides AA. Spontaneous dog osteoarthritis—a one medicine vision. Nat. Rev. Rheumatol. (2019) 15:273–287. 10.1038/s41584-019-0202-1 30953036 PMC7097182

[16] Bannuru RR Osani MC Vaysbrot EE Arden NK Bennell K Bierma-Zeinstra SMA. OARSI guidelines for the non-surgical management of knee, hip, and polyarticular osteoarthritis. Osteoarthr. Cartilage. (2019) 27:1578–89. 10.1016/j.joca.2019.06.011 31278997

[17] Park KD Kim TK Bae BW Ahn J Lee WY Park Y. Ultrasound guided intra-articular ketorolac versus corticosteroid injection in osteoarthritis of the hip: a retrospective comparative study. Skeletal. Radiol. (2015) 44:1333–40. 10.1007/s00256-015-2174-9 26031217

[18] NICE Osteoarthritis: care and management. NICE Guideline (2020). 33705083

[19] Kolasinski SL Neogi T Hochberg MC Oatis C Guyatt G Block J Arthritis Care Res. (2019) 72:220–33. 10.1002/acr.24131

[20] Bruyère O Honvo G Veronese N Arden NK Branco J Curtis EM. An updated algorithm recommendation for the management of knee osteoarthritis from the European Society for Clinical and Economic Aspects of Osteoporosis, Osteoarthritis and Musculoskeletal Diseases (ESCEO). Semin. Arthritis Rheum. (2019) 49:337–50. 10.1016/j.semarthrit.2019.04.008 31126594

[21] Jevsevar DS. Treatment of osteoarthritis of the knee: evidence-based guideline, 2nd Edition. J. Am. Acad. Orthop. Surg. (2013) 21:571–6. 10.5435/JAAOS-21-09-571 23996988

[22] Sellam J Berenbaum F. The role of synovitis in pathophysiology and clinical symptoms of osteoarthritis. Nat. Rev. Rheumatol. (2010) 6:625–35. 10.1038/nrrheum.2010.159 20924410

[23] Lavelle W Lavelle ED Lavelle L. Intra-articular injections. Anesthesiol. Clin. (2007) 25:853–62. 10.1016/j.anclin.2007.07.002 18054149

[24] Caron JP. Intra-articular injections for joint disease in horses. Vet. Clin. North Am. Equine Pract. (2005) 21:559–73. 10.1016/j.cveq.2005.07.003 16297721

[25] Vaishya R Pandit R Agarwal AK Vijay V. Intra-articular hyaluronic acid is superior to steroids in knee osteoarthritis: a comparative, randomized study. J. Clin. Orthop. Trauma. (2017) 8:85–8. 10.1016/j.jcot.2016.09.008 28360505 PMC5359523

[26] Céleste C Ionescu M Poole AR Laverty S. Repeated intraarticular injections of triamcinolone acetonide alter cartilage matrix metabolism measured by biomarkers in synovial fluid. J. Orthop. Res. (2005) 23:602–10. 10.1016/j.orthres.2004.10.003 15885481

[27] Garg N Perry L Deodhar A. Intra-articular and soft tissue injections, a systematic review of relative efficacy of various corticosteroids. Clin. Rheumatol. (2014) 33:1695–706. 10.1007/s10067-014-2572-8 24651914

[28] Colen S van den Bekerom MP Bellemans J Mulier M. Comparison of intra-articular injections of hyaluronic acid and corticosteroid in the treatment of osteoarthritis of the hip in comparison with intra-articular injections of Bupivacaine. Design of a prospective, randomized, controlled study with blinding of the patients and outcome assessors. BMC Musculoskelet Disord. (2010) 11:264. 10.1186/1471-2474-11-264 21080920 PMC2998460

[29] Evans CH. Novel biological approaches to the intra-articular treatment of osteoarthritis. BioDrugs. (2005) 19:355–62. 10.2165/00063030-200519060-00003 16392888

[30] Strauss EJ Hart JA Miller MD Altman RD Rosen JE. Hyaluronic acid viscosupplementation and osteoarthritis. Am. J. Sports Med. (2009) 37:1636–44. 10.1177/0363546508326984 19168804

[31] Sundman EA Cole BJ Karas V Della Valle C Tetreault MW Mohammed HO. The anti-inflammatory and matrix restorative mechanisms of platelet-rich plasma in osteoarthritis. Am. J. Sports Med. (2014) 42:35–41. 10.1177/0363546513507766 24192391

[32] Van Vynckt D Samoy Y Mosselmans L Verhoeven G Verschooten F Van Ryssen B The use of intra-articular anesthesia as a diagnostic tool in canine lameness. Vlaams Diergeneeskd Tijdschr. (2012) 81:290–7.

[33] Spadari A Rinnovati R Babbini S Romagnoli N Clinical evaluation of intra-articular administration of stanozolol to manage lameness associated with acute andchronic osteoarthritis in horses. J. Equine Vet. Sci. (2015) 35:105–10. 10.1016/j.jevs.2014.12.003

[34] Popma JW Snel FW Haagsma CJ Brummelhuis-Visser P Oldenhof HGJ van der Palen J. Comparison of 2 dosages of intraarticular triamcinolone for the treatment of knee arthritis: results of a 12-week randomized controlled clinical trial. J. Rheumatol. 42:1865–8. 10.3899/jrheum.141630 26233499

[35] Franklin SP Cook JL. Prospective trial of autologous conditioned plasma versus hyaluronan plus corticosteroid for elbow osteoarthritis in dogs. Can. Vet. J. (2013) 54:881–4. 24155495 PMC3743576

[36] Suntiparpluacha M Tammachote N Tammachote R. Triamcinolone acetonide reduces viability, induces oxidative stress, and alters gene expressions of human chondrocytes. Eur. Rev. Med. Pharmacol. Sci. (2016) 20:4985–92. 27981533

[37] Labens R Mellor DJ Voûte LC. Retrospective study of the effect of intra-articular treatment of osteoarthritis of the distal tarsal joints in 51 horses. Vet. Rec. 161:611–6. 10.1136/vr.161.18.611 17982139

[38] Vandeweerd J-M Zhao Y Nisolle J-F Zhang W Zhihong L Clegg P. Effect of corticosteroids on articular cartilage: have animal studies said everything? Fundam. Clin. Pharmacol. (2015) 29:427–38. 10.1111/fcp.12137 26211421

[39] Leardini G Mattara L Franceschini M Perbellini A. Intra-articular treatment of knee osteoarthritis. A comparative study between hyaluronic acid and 6-methyl prednisolone acetate. Clin. Exp. Rheumatol. (1991) 9:375–81. 1934686

[40] Rezende MU Andrusaitis FR Silva RT Okazaki E Carneiro JDA Campos GC Joint lavage followed by viscosupplementation and triamcinolone in patients with severe haemophilic arthropathy: objective functional results. Haemophilia. (2017) 23:e105–15. 10.1111/hae.13115 27860135

[41] Kuroki K Cook JL Kreeger JM. Mechanisms of action and potential uses of hyaluronan in dogs with osteoarthritis. J. Am. Vet. Med. Assoc. (2002) 221:944–50. 10.2460/javma.2002.221.944 12369696

[42] Felson DT Niu J Guermazi A Roemer F Aliabadi P Clancy M. Correlation of the development of knee pain with enlarging bone marrow lesions on magnetic resonance imaging. Arthritis Rheum. (2007) 56:2986–92. 10.1002/art.22851 17763427

[43] Canapp SO Cross AR Brown MP Lewis DD Hernandez J Merritt KA Examination of synovial fluid and serum following intravenous injections of hyaluronan for the treatment of osteoarthritis in dogs. Vet. Comp. Orthop. Traumatol. (2005) 18:169–74. 10.1055/s-0038-1632949 16594448

[44] Upchurch DA Renberg WC Roush JK Milliken GA Weiss ML. Effects of administration of adipose-derived stromal vascular fraction and platelet-rich plasma to dogs with osteoarthritis of the hip joints. Am. J. Vet. Res. (2016) 77:940–51. 10.2460/ajvr.77.9.940 27580105

[45] Murray RC Znaor N Tanner KE DeBowes RM Gaughan EM Goodship AE. The effect of intra-articular methylprednisolone acetate and exercise on equine carpal subchondral and cancellous bone microhardness. Equine Vet. J. (2010) 34:306–10. 10.2746/042516402776185994 12108753

[46] Carter BG Bertone AL Weisbrode SE Bailey MQ Andrews JM Palmer JL. Influence of methylprednisolone acetate on osteochondral healing in exercised tarsocrural joints of horses. Am. J. Vet. Res. (1996) 57:914–22. 8725823

[47] Pelletier J-P Martel-Pelletier J. Protective effects of corticosteroids on cartilage lesions and osteophyte formation in the pond-nuki dog model of osteoarthritis. Arthritis Rheum. (1989) 32:181–93. 10.1002/anr.1780320211 2920053

