# Supplementary material for: A Pilot Study on the Efficacy of a Single Intra-Articular Administration of Triamcinolone Acetonide, Hyaluronan, and a Combination of Both for Clinical Management of Osteoarthritis in Police Working Dogs
Source: Front Vet Sci. 2020 Nov 6;7:512523. doi: 10.3389/fvets.2020.512523 (PMC7690322; doi:10.3389/fvets.2020.512523)
Supplement: Supplementary file 2 [file Data_Sheet_2.PDF]

APPENDIX B – HUDSON VISUAL ANALOGUE SCALE

Hudson Visual Analogue Scale

1. How would you describe your **overall assessment** of your dog in the **last month**? (0-10, 0 = bad, 10 = good)
2. What kind of **mood** has your dog been in the last month? (0-10, 0 = bad, 10 = good)
3. How has your dog' s attitude been in the last month? (0-10, 0 = bad, 10 = good)
4. How frequently does your dog display comfort or "happy dog" postures (e.g., lying on back with toy in mouth? (0-10, 0 = bad, 10 = good)

Tell us what type of daily activities your dog engages in (e.g., fetching newspapers, playing frisbee) and then answer question 5.

5. Has your dog **changed the amount** of these activities? (0-10, 0 = not at all, 10 = a lot)
6. How willing is your dog to **play voluntarily**? (0-10, 0 = not at all, 10 = a lot)
7. How **often** does your dog get **exercise**? (0-10, 0 = not at all, 10 = a lot)

8. How **stiff** is your dog when **arising for the day** (0-10, 0 = not at all, 10 = a lot)

9. How **stiff** is your dog **at the end of the day** (post-activities)? (0-10, 0 = not at all, 10 = a lot)

10. Does your dog indicate any **lameness at a walk**? (0-10, 0 = not at all, 10 = a lot)

11. Does your dog indicate any **pain when turning suddenly at a walk**? (0-10, 0 = not at all, 10 = a lot)
